# Current status of premature mortality from four non-communicable diseases and progress towards the Sustainable Development Goal target 3.4: a population-based study in northeast China, 2004–2017

**DOI:** 10.1186/s12889-021-11611-0

**Published:** 2021-09-02

**Authors:** Li Liu, Yanxia Li, Junmin Song, Qian Chen, Shuang Li, Huijuan Mu, Jun Na, Rui Zhang, Liya Yu, Wei Sun, Guowei Pan

**Affiliations:** 1 Institute of Preventive Medicine, China Medical University, Shenyang, 110122 People’s Republic of China; 2 Institute of Chronic Diseases, Liaoning Provincial Center for Disease Control and Prevention, Shenyang, People’s Republic of China; 3 Department of Gastroenterology, Shengjing Hospital of China Medical University, Shenyang, 110004 People’s Republic of China; 4 Research Center for Universal Health, School of Public Health, China Medical University, No.77 Puhe Road, Shenyang North New Area, Shenyang, 110122 China

**Keywords:** Average annual percentage change, China, Chronic disease, Liaoning, Mortality, Non-communicable disease, Premature, Sustainable Development Goal

## Abstract

**Background and aim:**

According to the United Nations’ Sustainable Development Goal (SDG) target 3.4, premature mortality from four non-communicable diseases (cardiovascular diseases, cancer, chronic respiratory diseases, and diabetes mellitus, collectively referred to as NCD4) should achieve a minimum decline of 33% in 2030 relative to 2015. This remains a challenge for China. This study aimed to evaluate the current status and progress towards this target in Liaoning Province, one of the three provinces in northeast China.

**Methods:**

We calculated the premature mortality rates (PMRs) per year and average annual percentage changes (AAPCs) from NCD4 using mortality data between 2004 and 2017. The trend was analyzed in the whole population, as well as in subpopulations of gender (male/female) and inhabiting area (urban/rural). PMRs from NCD4 for 2030 were projected by fitting a linear regression based on the current trend, which was identified by a Joinpoint model.

**Findings:**

In the whole population, only chronic respiratory diseases showed a significant decline (AAPC: − 6.5%, *p* < 0.05), while only cancer showed a significant increase (AAPC: + 1.3%, *p* < 0.05); taken together, NCD4 showed a significant increase (AAPC: + 0.6%, *p* < 0.05). In the subpopulations, while males showed a significant increase in NCD4 (AAPC: + 1.5%, *p* < 0.05), cardiovascular diseases (AAPC: + 1.7%, *p* < 0.05), cancer (AAPC: + 1.8%, *p* < 0.05), and diabetes mellitus (AAPC: + 4.2%, *p* < 0.05), females showed a significant decline in NCD4 (AAPC: − 1.2%, *p* < 0.05), cardiovascular diseases (AAPC: − 1.8%, *p* < 0.05), diabetes mellitus (AAPC: − 2.1%, *p* < 0.05), but showed a mild increase in cancer (AAPC: + 0.5%, *p* > 0.05). A comparative analysis of the projected PMRs for 2030 with the 2015 levels revealed that only chronic respiratory diseases are expected to achieve the SDG target 3.4, apart from in the urban male subpopulation.

**Conclusion:**

Except for chronic respiratory diseases, NCD4 cannot be expected to achieve the SDG target 3.4 in the whole population of Liaoning Province. Under these circumstances, special attention should be paid to reducing the risks of cancer and providing preventative interventions for men.

## Background

Over the last century, the major threats to human health have undergone a transition from infectious to non-communicable diseases (NCDs). The latter is nowadays increasingly becoming a tremendous mortality burden, accounting for up to 71% of all deaths worldwide, especially in low- and middle-income countries [1, 2]. Among NCDs, four diseases have gained special attention due to their prevalence and mortality: cardiovascular diseases (CVDs), cancer, chronic respiratory diseases (CRDs), and diabetes mellitus (DM), which are collectively referred to as NCD4. NCD4 may be responsible for deaths in various age groups, but those occurring in patients between 30 and 70 years are defined as premature mortality. Over the years, much effort has been paid to reducing global premature mortality from NCD4. In 2012, the World Health Assembly planned to reduce premature mortality from NCD4 by a minimum of 25% by 2025, relative to the 2010 level (e.g., 25 × 25 target) [3]. More recently, the United Nations General Assembly released a 2030 Agenda of Sustainable Development Goals (SDGs), which announced the new target of a minimum 33% decline relative to 2015 levels (e.g., SDG target 3.4, available from http://www.un.org/sustainabledevelopment/health/). In practice, this target remains a challenge for many countries worldwide. It has been estimated that only 35 countries (19% of all countries) will likely achieve the target [1]. Meanwhile, the Chinese central government also set a target of at least a 30% decline in 2016 [4], which translates to a decline in the NCD4 premature mortality rate from 19.1 to 13.4% in China (Healthy China 2030), and from 17.0 to 11.9% in Liaoning Province (Healthy Liaoning 2030). Although both the central and local governments in China have provided substantial support for this target, problems remain. In a study that assessed the progress of Healthy China 2030 from 1990 through 2015, the premature mortality rate showed a 2.0% decline per year on average; thus, all females but only males in 11 out of 33 provinces would be expected to achieve the Chinese target [4]. However, according to a projection study, China can possibly achieve the target if appropriate interventions against risk factors are performed [5]. Despite a series of native publications that were focused on this topic [4, 6–8], thus far, there have been few reports on the status and progress of northeast China towards these health goals.

In this study, we first evaluate the current status in Liaoning Province, and then analyze whether SDG target 3.4 can be achieved in the whole population as well as in the subpopulations of gender and inhabiting area. The latter analysis may warrant intensive surveillance and active interventions for the subpopulations most at risk of not achieving SDG target 3.4.

## Methods

### Study population

Mortality data between 2004 and 2017 were retrieved from the Vital Registry System of Liaoning Provincial Center for Disease Control and Prevention (LNCDCP). Liaoning Province is located in the northeast of China, encompassing 14 cities and 44 counties. A population-based vital registry system for Liaoning Province has been established in the cities since 1982 and in the counties since 2000. The registry system is completed and maintained at three levels. First, doctors in all hospitals and community health service centers are responsible for determining cause of death, completing death certificates, and coding events according to the 10th revision of the international classification of diseases (ICD-10). Second, the public health staff in the city or county Center for Disease Control and Prevention are responsible for overseeing the daily reported deaths and verifying medical certificates for all deaths that occurred outside of hospitals/community centers to minimize cases with missing reports and unknown causes of death. In cases of incomplete or low-quality reporting, the certificates are returned and re-completed. Third, LNCDCP provides technical training and performs quality control to ensure completeness, consistency, and data quality. To make a reliable assessment of the NCD4 trend, we used the mortality data from seven cities (11.3 million residents) and 10 counties (4.8 million residents), covering 49.5 and 25.0% of the urban and rural populations, respectively. In our province, only death registry data from these cities and counties have been qualified by the Chinese Center for Disease Control and Prevention and included in the national death registry database. In general, the crude mortality rates were 615.51–892.43 per 10^5^, and the proportion of unknown causes of death were 0.12–1.53% for these cities and counties between 2004 and 2017. Thus, both met the criteria of the Chinese Guideline for Death Registry (e.g., crude mortality is typically > 600 per 10^5^and the proportion of unknown causes of death is typically < 5%).

### Statistical analysis

We set premature mortality rate (PMR) as the primary analytical parameter for this study. PMR is defined as unconditional probability of death among those aged between 30 and 70 years from any component of the NCD4.

First, yearly PMRs for each of the four diseases were calculated. The formulae and calculation steps are described in Noncommunicable Diseases Progress Monitor 2020 by The World Health Organization (available fromhttps://www.who.int/publications/i/item/ncd-progress-monitor-2020), and a simplified set of procedures are presented in the following three steps. SPSS 19.0 (IBM Corp., Armonk, NY, USA) was used for all calculations.

Step 1: Age-specific mortality rates in 5-year age groups were calculated:

$$ {}_5{}^{\ast }{M}_x=\frac{Total\ deaths\ from\ four\ NCDs\ aged\ \left[x,x+5\right)}{Total\ population\ aged\ \left[x,x+5\right)} $$

Step 2: The 5-year mortality rate ${}_5{}^{\ast }{M}_x$ was translated into probability of mortality:

$$ {}_5{}^{\ast }{q}_x=\frac{{}_5{}^{\ast }{M}_x\ast 5}{1+{}_5{}^{\ast }{M}_x\ast 2.5} $$

Step 3: The unconditional probability of mortality among persons aged between 30 and 70 years was calculated:

$$ {}_{40}{}^{\ast }{q}_{30}=1-\prod \limits_{x=30}^{65}\left(1-{}_5{}^{\ast }{q}_x\right) $$

Second, we used the Joinpoint Regression Program (version 4.8.0.1; US National Cancer Institute, Bethesda, MD, USA) to estimate the annual percentage change (APC) and average APC (AAPC). These formulae are presented as follows:

$$ {APC}_i=\left\{\exp \left({b}_i\right)-1\right\}\times 100 $$

$$ AAPC=\left\{\exp \left(\frac{\sum {w}_i{b}_i}{\sum {w}_i}\right)-1\right\}\times 100 $$

In these formulae, *b*_*i*_ refers to the slope coefficients for each segment in the years studied, and *w*_*i*_ refers to the length of each segment in the intervals. The one-third reduction requested by SDG target 3.4 can be translated into a minimum AAPC of − 2.22%; that is to say, AAPC ≤ − 2.22% is required to achieve the target.

Finally, PMRs from NCD4 for 2030 were projected by fitting a linear regression based on the current trend, which was identified by the Joinpoint model. This model used the PMRs of NCD4 to fit a log linear model based on a Poisson distribution. The number of connection points, the location of each connection point, and the corresponding *p* values were determined by Monte Carlo permutation test. The best model was set according to Bayesian information criterion. In this study, the model parametric setting allowed up to two connection points, and a *p* value of less than 0.05 indicated that the difference between connection points was statistically significant (Joinpoint Help Manual 4.8.0.1, available from https://surveillance.cancer.gov/help/joinpoint/).

## Results

**Fig. 1:**
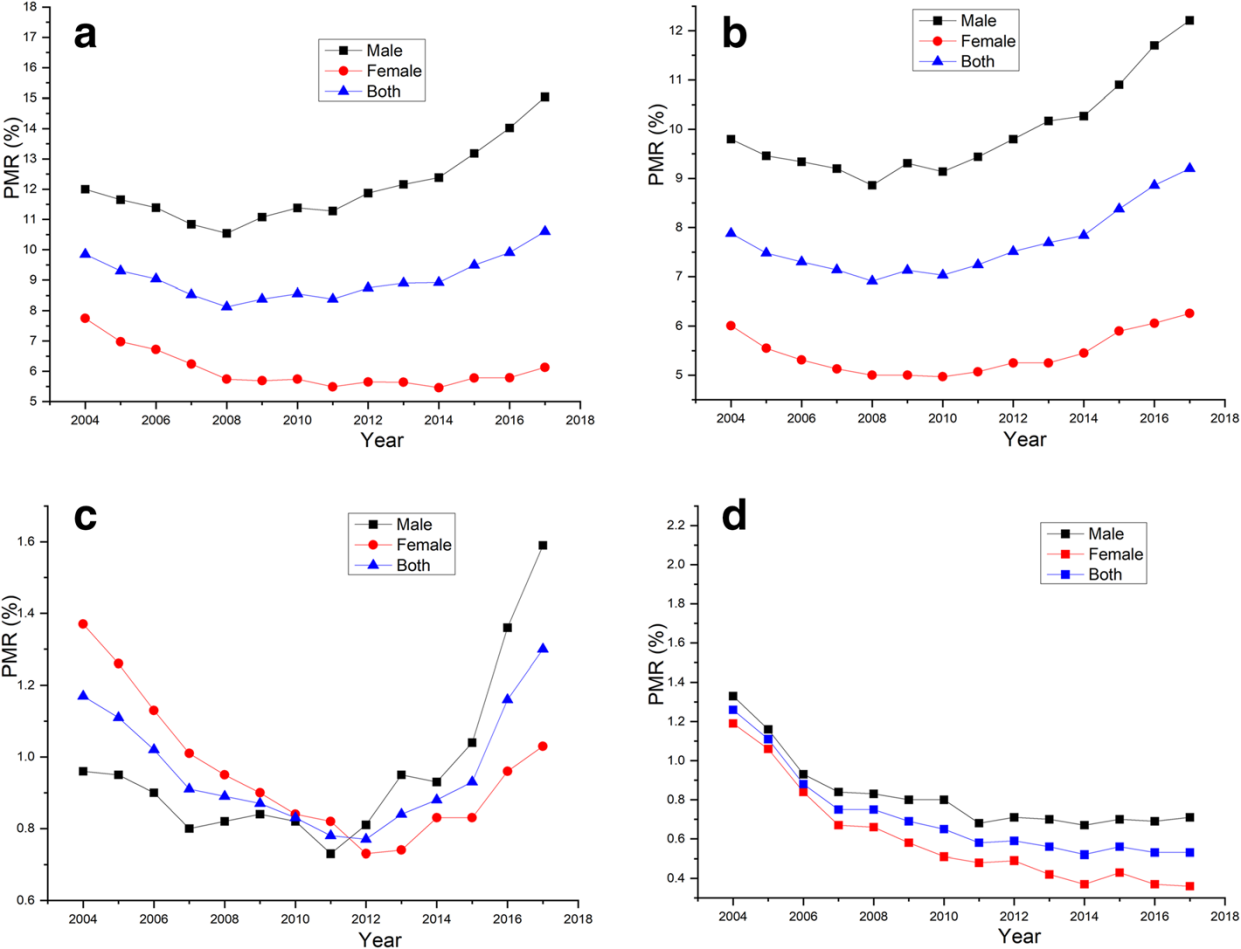
Dynamic premature mortality from components of the NCD4 over time from 2004 through 2017 in both genders: **a**, CVDs; **b**, cancer; **c**, DM; **d**, CRDs

**Fig. 2:**
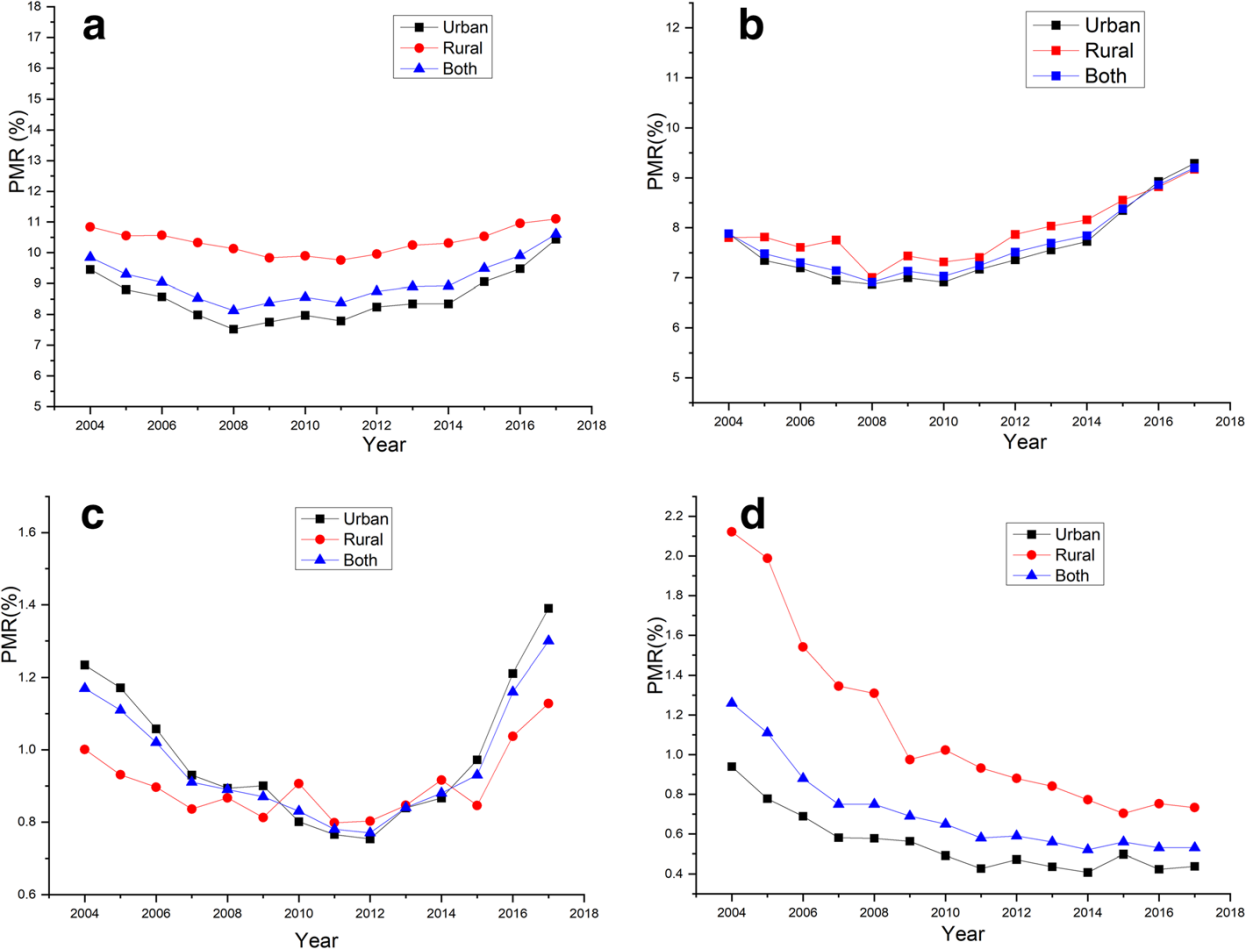
Dynamic premature mortality from components of the NCD4 over time from 2004 through 2017 in both inhabiting areas: **a**, CVDs; **b**, cancer; **c**, DM; **d**, CRDs

First, the dynamics of premature mortality over the 14-year study period in subpopulations of gender and inhabiting area are presented in Figs. 1 and 2. As shown in Fig. 1 (grouping by gender), all diseases but CRDs showed a U-shaped change, with the bottom being 2008–2012. Remarkable gaps were noted in CVDs and cancer, but not DM, which showed some overlap between the genders; CRDs showed a continuous decline in both genders. As shown in Fig. 2 (grouping by inhabiting areas), all diseases but CRDs showed a U-shaped change, with the bottom also being 2008–2012. For CVDs, the rural population always maintained a higher level than urban inhabitants, with less remarkable gaps; for cancer and DM, minor gaps and some overlaps were noted between both inhabiting areas. Lastly, CRDs showed a continuous decline in both inhabiting areas.

Second, the PMRs per year and AAPC (%) of the 14 years in the whole population, as well as in the subpopulations of gender and inhabiting area are presented in Table 1. In the whole population, only CRDs showed a significant decline (AAPC: − 6.5%, *p* < 0.05), while only cancer showed a significant increase (AAPC: + 1.3%, *p* < 0.05). Together, NCD4 showed a significant increase (AAPC: + 0.6%, *p* < 0.05). Among the subpopulations, only CRDs showed a consistent decline, with AAPCs ranging between − 3.8% (urban males, *p* < 0.05) and − 9.9% (rural females, *p* < 0.05); conversely, only cancer showed a consistent increase, with AAPCs ranging between + 0.5% (urban females, *p* > 0.05) and + 2.1% (urban males, *p* < 0.05). Both CVDs and DM showed bidirectional AAPC results among the subpopulations (+ 2.1% and + 4.8% for CVDs and DM in urban males, respectively; − 2.2% and − 3.0% for CVDs and DM in urban females, respectively; all *p* < 0.05). Similar to CVDs and DM, NCD4 also showed bidirectional AAPC results, ranging between − 1.3% (urban females, *p* < 0.05) and + 1.9% (urban males, *p* < 0.05). Inequalities were mainly observed between the genders, and to a much lesser extent, between inhabiting areas. Males showed a significant increase in NCD4 (AAPC: + 1.5%, *p* < 0.05), CVDs (AAPC: + 1.7%, *p* < 0.05), cancer (AAPC: + 1.8%, *p* < 0.05), and DM (AAPC: + 4.2%, *p* < 0.05). In contrast, females showed a significant decline in NCD4 (AAPC: − 1.2%, *p* < 0.05), CVDs (AAPC: − 1.8%, *p* < 0.05), DM (AAPC: − 2.1%, *p* < 0.05), but a mild increase in cancer (AAPC: + 0.5%, *p* > 0.05). Urban males showed higher AAPCs (+ 1.9%, + 2.1%, + 2.1%, + 4.8%, for NCD4, CVDs, cancer, and DM, respectively; all *p* < 0.05) than rural males.

**Table 1:** AAPC analysis of premature mortality rate (%) for NCD4

	Male			Female			Male+ Female		
	Urban	Rural	Urban+Rural	Urban	Rural	Urban+Rural	Urban	Rural	Urban+Rural
NCD4									
2004	21.96	23.58	22.44	14.93	16.93	15.50	18.40	20.35	18.97
2017	27.84	25.89	27.14	12.44	15.31	13.23	20.25	20.76	20.32
AAPC (%)	1.9*	0.7*	1.5*	−1.3*	−0.6	−1.2*	0.8*	0.1	0.6*
95% CI	(1.2, 2.6)	(0.4, 1.0)	(0.9, 2.1)	(−2.3, −0.3)	(−1.4, 0.1)	(−1.8, − 0.5)	(0.2, 1.5)	(− 0.2, 0.3)	(0.0, 1.1)
CVDs									
2004	11.68	12.77	12.00	7.31	8.83	7.75	9.46	10.84	9.85
2017	15.48	14.25	15.04	5.44	7.86	6.13	10.44	11.10	10.60
AAPC (%)	2.1*	0.9*	1.7*	−2.2*	−1.0*	−1.8*	0.7	0.2	0.6
95% CI	(1.3, 2.9)	(0.5, 1.3)	(1.1, 2.3)	(−3.5, −0.9)	(−1.7, −0.3)	(−2.7, −0.8)	(−0.2, 1.6)	(0.0, 0.5)	(−0.1, 1.2)
Cancer									
2004	9.78	9.80	9.80	6.09	5.72	6.01	7.88	7.80	7.88
2017	12.46	11.85	12.21	6.25	6.43	6.26	9.29	9.17	9.20
AAPC (%)	2.1*	1.2*	1.8*	0.5	0.8	0.5	1.5*	1.2*	1.3*
95% CI	(1.1, 3.1)	(0.5, 1.9)	(1.0, 2.6)	(−0.3, 1.3)	(−0.2, 1.9)	(−0.3, 1.4)	(0.8, 2.3)	(0.5, 1.9)	(0.6, 2.0)
Diabetes mellitus									
2004	1.05	0.73	0.96	1.39	1.28	1.37	1.23	1.00	1.17
2017	1.85	1.05	1.59	0.96	1.21	1.03	1.39	1.13	1.30
AAPC (%)	4.8*	2.6	4.2*	−3.0*	−0.2	−2.1*	0.8	1.0	0.7
95% CI	(0.7. 9.0)	(−0.2, 5.5)	(0.8, 7.6)	(−4.1, −1.9)	(−2.2, 1.9)	(− 3.2, −1.1)	(− 0.6, 2.2)	(− 0.9, 2.8)	(−0.6, 2.0)
CRDs									
2004	1.01	2.14	1.33	0.87	2.10	1.19	0.94	2.12	1.26
2017	0.61	0.90	0.71	0.28	0.56	0.36	0.44	0.73	0.53
AAPC (%)	−3.8*	−7.0*	−5.0*	−8.1*	−9.9*	−8.8*	−5.0*	−8.5*	−6.5*
95% CI	(−6.2, −1.4)	(−9.1, −4.9)	(−6.2, − 3.9)	(− 10.9, −5.1)	(−11.6, −8.3)	(− 10.7, − 7.0)	(−7.0, −2.9)	(−10.0, − 6.9)	(−8.1, − 4.8)

*Abbreviations*: *AAPC* average annual percentage change, *CI* confidence interval, *CRD* chronic respiratory disease, *CVD* cardiovascular disease, *NCD4* four non-communicable diseases

*P < 0.05

Third, the AAPCs of some individual diseases within CVDs and cancer are presented in Table 2. Among the whole population, heart diseases showed a significant increase (AAPC: + 2.5%, *p* < 0.05), stroke showed a mild decline (AAPC: − 0.8%, *p* < 0.05); and most cancers were increasing, with AAPCs ranging between − 3.0% (bone cancer, *p* > 0.05) and + 8.5% (cervical cancer, *p* < 0.05). Among the subpopulations, bidirectional AAPCs were observed in heart diseases (ranging between − 0.4% [urban females, *p* > 0.05] and + 4.1% [urban males, *p* < 0.05]), stroke (ranging between − 3.9% [urban females, *p* < 0.05] and + 0.7% [urban males, *p* > 0.05]), and most individual cancers. In total, inequalities were mainly observed between the genders, and to a much less extent, between inhabiting areas, with a few exceptions. While males showed an increase in heart diseases (AAPC: + 3.8%, *p* < 0.05), stroke (AAPC: + 0.4%, *p* > 0.05), and most cancers, females showed no change in heart diseases (AAPC: 0, *p* > 0.05), a decline in stroke (AAPC: − 3.3%, *p* < 0.05), and some cancers. Urban males showed higher AAPCs for heart diseases (+ 4.1%, *p* < 0.05), and cancers of the lung, liver, colon/rectum, esophagus, and lymphoma(+ 2.3%, + 1.3%, + 4.9%, + 1.9%, and + 3.1%, respectively; all *p* < 0.05) compared with rural males.

**Table 2:** AAPC analysis of premature mortality rate (%) for some subsets of NCD4

Disease	ICD	Male			Female			Male+ Female		
		Urban	Rural	Urban+Rural	Urban	Rural	Urban+Rural	Urban	Rural	Urban+Rural
CVDs										
Heart diseases	I05-I09, 11, 20–27, 30–52	4.1*	3.4*	3.8*	−0.4	1.2*	0.0	2.6*	2.6*	2.5*
Stroke	I60–69	0.7	−0.4*	0.4	−3.9*	−2.5*	− 3.3*	−0.8	−1.2*	− 0.8*
Cancer										
Lung	C33–34	2.3*	2.0*	2.0*	−1.7*	1.3*	−1.2*	0.9*	1.4*	0.9*
Liver	C22	1.3*	0.9	1.0*	0.5	0.3	0.5	1.1*	0.7	0.9*
Stomach	C16	0.6	−1.0	−0.5	−2.4*	−2.7*	−2.3*	− 0.2	− 2.0*	−1.1
Colon–rectum	C18–21	4.9*	4.5*	4.7*	0.6	2.5*	2.0	3.4*	3.8*	3.2*
Breast	C50	..	..	..	2.9*	2.3*	2.8*	2.8*	2.3*	2.8*
Pancreas	C25	2.2*	3.6*	2.4*	3.0*	5.3*	3.1*	2.4	4.1*	2.5*
Esophageal	C15	1.9*	0.0	1.4*	−10.5*	−2.7	−6.0*	1.1	−0.4	0.3
Cervix	C53	..	..	..	7.3*	8.5*	8.5*	7.3*	8.6*	8.5*
Ovary	C56	..	..	..	2.9*	8.9*	4.7*	3.0*	9.3*	4.5*
Brain	C70–72	1.5	0.4	1.2	−1.3	0.4	−0.9	0.5	0.4	−0.1
Gallbladder	C23–24	2.3*	4.6*	2.3*	−1.5	3.4*	0.0	0.8	3.9*	1.3
Kidney	C64–66, C68	4.9*	7.7*	4.8*	2.1	9.8*	3.8*	4.6*	8.5*	4.4*
Lymphoma	C81–85, C88, C90, C96	3.1*	−1.0	1.4	6.2*	−3.0	1.4	4.2*	−2.2	1.2
leukemia	C91–95	1.5	2.4*	1.8*	1.2	1.8	1.5*	1.3	2.0*	1.8*
Larynx	C32	2.5	2.7	2.9	..	..	..	1.5	1.1	1.7
Bladder	C67	1.3	0.8	1.6	−4.2	−1.3	−3.4	−0.3	0.0	0.6
Bone	C40–41	−3.1	1.1	−0.8	−4.2	−0.7	−3.8	−3.0	1.6	−3.0
Uterus	C54-C55	..	..	..	1.9	−3.9*	−0.2	1.7	−4.1*	−0.1
Nasopharynx	C11	0.8	3.1	1.6	−2.0	2.0	−1.6	−0.5	3.2	0.1

*Abbreviations*: *AAPC* average annual percentage change, *CVD* cardiovascular disease, *NCD4* four non-communicable diseases

*: *P* < 0.05

Finally, given that the PMRs of CVDs, cancer, and DM after 2012, and the PMRs of CRDs after 2007 showed a relatively more linear trend (Figs. 1 and 2), we used the mortality data of CVDs, cancer, and DM between 2012 and 2017, and the mortality data of CRDs between 2007 and 2017 to generate 2030 projections. A comparative analysis of the PMRs projected for 2030 with the 2015 levels is presented in Table 3. Urban males were the only subpopulation that were not predicted to achieve the SDG target 3.4 for CRDs. None of the other three diseases can be expected to achieve the target in either the whole population or in subpopulations.

**Table 3:** Comparison of premature mortality rate (%) projected for 2030 with the 2015 level

	Urban + Rural		Urban		Rural	
	2030	2015	2030	2015	2030	2015
Male						
NCD4	47.91 (37.55,61.84)	24.00	54.48 (38.60, 77.67)	23.87	37.57 (34.42, 41.00)	24.40
CVDs	27.42 (20.79, 36.01)	13.18	30.97 (19.46, 48.77)	12.99	21.53 (18.35, 25.56)	13.65
Cancer	21.71 (17.31, 27.54)	10.90	25.10 (18.78, 32.96)	10.85	16.98 (13.98, 20.90)	11.11
DM	8.15 (2.96, 22.38)	1.04	11.46 (3.69, 35.81)	1.18	3.49 (1.35, 8.97)	0.72
CRDs	0.50* (0.39, 0.65)	0.70	0.48* (0.33, 0.67)	0.67	0.48** (0.28, 0.85)	0.78
Female						
NCD4	18.47 (14.40, 23.93)	12.46	19.13 (13.58, 26.78)	11.78	18.49 (14.43, 23.29)	14.18
CVDs	7.34 (5.23, 10.25)	5.78	7.05 (4.75, 10.49)	5.17	8.24 (5.85, 11.54)	7.29
Cancer	10.44 (8.30, 13.25)	5.9	10.82 (7.89, 14.94)	5.92	10.34 (8.83, 12.27)	5.94
DM	2.55 (1.65, 4.01)	0.83	3.09 (1.74, 5.50)	0.77	2.00 (0.95, 4.13)	0.97
CRDs	0.15** (0.11, 0.20)	0.43	0.12** (0.06, 0.24)	0.34	0.17** (0.12, 0.23)	0.62
Male +Female						
NCD4	33.32 (26.51, 43.02)	18.31	36.95 (27.01, 50.95)	17.87	28.09 (25.26, 31.74)	19.45
CVDs	17.02 (12.65, 22.80)	9.49	18.52 (12.20, 28.29)	9.06	14.73 (13.24, 16.36)	10.53
Cancer	16.03 (13.24, 19.68)	8.38	17.66 (13.87, 22.40)	8.34	13.70 (12.15, 15.51)	8.55
DM	4.85 (2.42, 9.60)	0.93	6.51 (3.29, 13.12)	0.97	2.50 (1.17, 5.43)	0.85
CRDs	0.30** (0.24, 0.39)	0.56	0.28** (0.18, 0.42)	0.50	0.29** (0.20, 0.42)	0.70

The projected PMRs for 2030 and 95% confidence interval are presented; *nearly achieving the target; **achieving the target

*Abbreviations*: *CRD* chronic respiratory disease, *CVD* cardiovascular disease, *DM* diabetes mellitus, *NCD4* four non-communicable diseases

## Discussion

In this study, we evaluated the current status of premature mortality from NCD4 and progress towards the SDG target 3.4 in Liaoning Province. Our main findings are summarized as follows: (1) dynamic premature mortality over time in all diseases except CRDs showed a U-shaped change with the bottom being 2008–2012; (2) except for CRDs, the target for NCD4 cannot be expected to be achieved in the whole population of Liaoning Province, especially due to the data on cancer, which showed a consistent increase, and thus, should be paid special attention; (3) inequalities were mainly observed between the genders and to a much lesser extent between inhabiting areas, for example, males, in particular urban males, were the most far offtrack. Taken together, the current health situation is extremely urgent, and we need to take actions that bring Liaoning Province on course to achieve SDG target 3.4. Four important implications that should be emphasized are discussed below.

First, the dynamics of premature mortality over time for all diseases except CRDs showed a U-shaped curve, with the bottom being 2008–2012. A similar finding was observed in Hunan Province, where excess mortality started from 2008 to 2009 [9]. The difference is that the premature mortality showed a continuous decline in Hunan, but first a decline, and then an increase since 2008–2012 in Liaoning. Neither finding is self-explanatory, and both deserve in-depth investigations. The pathogenesis and development of CVDs, DM, and cancer are driven by several long-standing risk factors, such as hypertension, metabolic syndrome, obesity, high-salt diet, inadequate physical exercise, air pollution, among others. Thus, the underlying reasons for increase in mortality from CVDs, DM, and cancer since 2008–2012 in Liaoning Province may actually date back to 10–20 years earlier, e.g., the 1990s. Since about a decade after the reform/opening-up policy was carried out, the Chinese population had been experiencing an unprecedented economic transition with 1990–1999 being the watershed decade. As an important indicator, the gross domestic product (GDP) per capita for every decade had increased by 1.62-fold for 1970–1979, 1.60-fold for 1980–1989, 2.75-fold for 1990–1999, and 4.00-fold for 2000–2009 (available from https://www.kylc.com/stats/global/yearly_per_country/g_gdp_per_capita/chn.html). Thus, the first thing to accompany increased income was inevitably food consumption, which in turn produced various alterations in behaviors, notions, environment, and lifestyles. Consistent with this explanation, food consumption began to dominate water, carbon, and ecological footprints during the last decade of the last century [10]. Specifically, egg consumption per capita underwent a remarkable increase within the period of 1990–1999 (the incremental fold-changes of the previous four decades being 1.21, 2.20, 2.42, and 1.20, respectively, available from http://www.fao.org/faostat/zh/?#compare). Previous studies have demonstrated that higher egg intake is associated with an increased risk of CVDs and DM [11, 12]. Briefly, factors that are a direct consequence of food consumption choices may interplay and collectively be responsible for the unfavorable increase in PMR since 2008–2012 in Liaoning Province.

Second, NCD4 encompasses CVDs, cancer, DM, and CRDs. These chronic non-communicable diseases can be assigned to two categories with CRDs alone in one category and the other three diseases in another category. Despite their non-communicable nature, infections play a key mechanistic role in the exacerbation and development of CRDs. Thus, because infections were being effectively controlled over the study period, the premature mortality from CRDs was likely to undergo a corresponding decline. For instance, in a previous study, we found that mortality from chronic obstructive pulmonary diseases showed a significant decline from 1984 through 2010 in Liaoning Province [13]. Rather than infections, metabolic disturbances underlie the pathogenesis and development of CVDs, DM, and at least some cancers [14, 15]. Thus, it is reasonable that both categories showed different trends. From the model used in this study, CRDs would be expected to completely achieve the SDG target 3.4 except for the subpopulation of urban males. However, the three other diseases would fail, primarily because it will likely take a long time for interventions against risk factors, such as smoking, alcohol abuse, unhealthy diets, overweight/obesity, increase blood pressure and glucose levels, less physical exercise, among others, which are still prevalent in Liaoning Province, to have meaningful effects. According to the findings of the China adult tobacco survey (available from http://www.chinacdc.cn/yw_9324/201905/t20190530_202932.html), the proportion of smokers in 2018 was 26.6% overall, and 50.5% for males over 15-years-old. Moreover, the mortality of lung cancer attributable to smoking in China during 1990–2017 showed an increasing trend [16]. The situation also remains serious for alcohol abuse. Among the 3 million global deaths attributable to alcohol consumption in 2016, CVDs accounted for 19.0%, and cancer accounted for 12.6% (Global status report on alcohol and health 2018, available from https://www.drugsandalcohol.ie/29701/). In Chinese adults aged over 18 years, the proportion of drinkers in 2010–2012 was 30.5% overall, and 53.8% in males [17]. In our province, a total of 5251 cancer deaths were attributable to alcohol consumption in 2012, accounting for 5.9% of all cancer deaths [18]. Additionally, the prevalence of high leisure sedentary time (≥4 h) increased by 58.58% from 2004 to 2011, and this unhealthy behavior can increase consumption of cigarettes and alcohol in addition to the risk of obesity [19]. More importantly, this finding reveals that the interventions carried out to date have been inadequate. Compared with CVDs and DM, which both showed a relative stagnation in mortality, cancer showed a remarkable increase in the whole population. Moreover, in 2017 Liaoning Province ranked as one of the top two and three provinces in China in terms of disease burden for colorectal and pancreatic cancer, respectively [20, 21]. Additionally, a recent survey showed that the combined 5-year age-standardized relative survival rate for 21 cancers diagnosed between 2000 and 2002 was as low as 41.5% in Liaoning Province [22]. Collectively, these findings highlight that more effort should be paid to ease the cancer burden in Liaoning Province.

Third, we examined differences in premature mortality from NCD4 in various subpopulations and mainly found inequalities between the genders, and to a much lesser extent between inhabiting areas. Except for DM, which showed some overlap between the genders, males always had higher levels of disease-related early mortality than females. A male predominance has also been documented in several native and international publications [4, 9, 23]. Underlying the predominance of these diseases in males worldwide is the higher prevalence of risk factors, such as smoking, alcohol abuse, hypertension, among others [24–28]. Notably, urban males were the most far offtrack from the goal. Although urban males are well educated, have a higher income, and better access to medical facilities, they also have more unhealthy diets, more stressful daily activities, less physical exercise, longer sedentary time, and heavier air pollution than rural males [29–31]. Consistently, hypertension, carotid atherosclerosis, and overweight/obesity all showed a predisposition for urban residents and males in China [24, 29, 32]. Additionally, only urban males were predicted to not achieve the SDG target 3.4 for CRDs, primarily due to more air pollution and a higher prevalence of smoking, both of which are key risk factors for CRDs [33, 34]. It is well known that in the past, urban and rural residents had great disparities with regards to economics, education, and medical facilities. According to the literature, lower economic conditions are associated with smoking, poor fruit and vegetable consumption, overweight/obesity, and inadequate physical exercise; lower educational achievement was associated with alcohol abuse [35]. Additionally, a previous study revealed that both pre-DM and DM were more prevalent among rural residents in Liaoning Province due to insufficient medical facilities and poor recognition [36]. Thus, different temporal trends may exist, and accordingly we hypothesized that rural inhabitants would be critically more urgent than urban residents. However, the urban-rural difference was less remarkable, with extreme examples being cancer and DM, indicating that the demarcation line between urban and rural is becoming obscure with the progress of urbanization courses, and thereby the risk factors exert a nearly equal impact on urban and rural communities. Nevertheless, it should be noted that in this study, the PMRs for CVDs, CRDs, and NCD4 were all at higher levels in rural residents than in urban communities. One of the main reasons for this finding is insufficient medical resources as a result of low household income in rural areas [37]. The urban-rural disparities exist in both medical facilities and workforce resources [38]. More medical facilities are available for urban than for rural residents [39, 40]. Additionally, there was a greater density of Chinese doctors and nurses in urban areas than in rural regions in 2005, in particular for nurses, for which the difference may exceed 3-fold [41]. Accordingly, a modest amount of effort is still required to address these diseases in rural areas. In this regard, the Chinese government has now been building up a strict hierarchical medical service system and encouraging more visits to doctors in local hospitals/clinics. Meanwhile, more medical students and free medical educational programs are being assigned to rural areas [42, 43]. These measures may prompt a switch of urban medical resources to rural areas, further narrowing the gap of premature mortality between these areas.

Fourth, the findings of our study warrant immediate and active interventions for the population of Liaoning Province, including tobacco/alcohol control, treatments for hypertension, CVDs, and DM, restrictions to high-salt diets, elimination of ambient particulate matter air pollution, and processes that will focus on the prevention, detection, and treatment of cancers [44, 45]. In particular, there should be a strong focus on tobacco use, high systolic blood pressure, high-salt diet, and ambient air pollution because these were the most common contributors to mortality in 2017 in China [46]. Despite the many interventions that have been executed or are in practice, continuous innovations by researchers and policymakers are greatly needed. For hypertension, DM, and cancer, large-scale population-wide screening should be conducted regularly because such patients are usually asymptomatic in early stages. Additionally, more effective diagnostic and therapeutic modalities should be utilized. Among the Chinese population with hypertension and DM, less than half were aware of their condition, and less than 40% had their blood pressure and glucose under control, respectively [47, 48]. Thus, awareness of these diseases and compliance with treatments should be improved [49, 50]. For tobacco/alcohol control, stricter laws/policies should be taken into consideration, especially with regards to juveniles, drunk driving, and indoor smoking, which should be strictly monitored by laws. As with many activities, industrialization and urbanization have unfavorable short- and long-term impacts on air pollution and the resultant diseases [51–53]. A system of surveillance and rewards/penalties should be established by law/policy. Energy combustion, illegal emissions, stagnant meteorological conditions, and biomass burning are main sources of haze formation in northeast China [54]. The Chinese government has proposed a policy advocating a transition from coal to natural gas/electricity in the northern provinces. As a result, both coal consumption and the emission of harmful gases have dropped remarkably in Liaoning Province from 2015 through 2019 as active responses to this policy [55]. Similar policies should be extended to other industries, such as manufacturing, processing, construction, and so on. A high-salt diet is prevalent in our province, and as an important factor for hypertension, CVDs and other diseases, it should be restricted [56, 57]. For instance, a low-sodium substitute remarkably reduced the risk of increased blood pressure in a selected population from Liaoning Province compared with regular salt [58]. Finally, lack of adequate physical exercise is a leading factor associated with overweight/obesity, hyperlipidemia, fatty liver, metabolic syndrome, and other conditions. Therefore, the entire population is encouraged to perform adequate physical exercises by various means. Last but not least, various education programs can be attached to the laws/policies that govern these interventions.

Finally, our study has strengths and limitations. The strengths include that the death registry data used in this population-based study have been qualified by the Chinese National Center for Disease Control and Prevention and included in the national death registry database, and thereby are authentic. By revealing the current status and progress, we can achieve the target soon after clearing the obstacles. The main limitation of this study is that it lacked a procedure for multiple-factor correction, which is because premature mortality is influenced by a variety of determinant factors, such as environment, behavior, and especially socioeconomic factors; however, data related to these factors were unavailable.

## Conclusions

The epidemiological features of NCD4 continue to evolve. With social and economic development, the previously existing urban/rural health disparities have largely narrowed. Instead, gender differences remain: males, particularly urban males, are the main obstacle to achieving the SDG target 3.4 and Chinese target in Liaoning Province. Thus, more policies and financial resources should be devoted to these subpopulations in addition to the whole population. Among the components of NCD4, cancer shows a continuous increase, and thus, should be paid special attention. Given that all three provinces in northeast China share similarities in social/economic development and lifestyles, the implications of our study may be applicable to neighboring provinces.

## Data Availability

The datasets generated and/or analyzed during the current study are not publicly available due to the Management Regulations on Death Data of LNCDCP but are available from the corresponding author on reasonable request.

## Abbreviations

AAPC: Average annual percentage change
APC: Annual percentage change
CRD: Chronic respiratory disease
CVD: Cardiovascular disease
DM: Diabetes mellitus
ICD: International Classification of Diseases
LNCDCP: Liaoning Provincial Center for Disease Control and Prevention
NCD4: Four non-communicable diseases
PM: Particulate matter
PMR: Premature mortality rate
SDG: Sustainable Development Goal
